# Bridging tradition and innovation: a review of computer simulations in plant breeding

**DOI:** 10.1007/s00122-026-05193-x

**Published:** 2026-06-09

**Authors:** Benjamin Stich, Delphine Van Inghelandt, Po-Ya Wu

**Affiliations:** 1 Federal Research Centre on Cultivated Plants, Institute for Breeding Research on Agricultural Crops, Julius Kuehn-Institute (JKI), Sanitz, 18190 Germany; 2 Professorship for Utilization of Plant Genetic Resources for Breeding Purposes, University of Rostock, Rostock, 18059 Germany; 3 Leibniz Institute of Plant Genetics and Crop Plant Research, Seeland, 06466 Germany

## Abstract

The wide range of tools and methods available to plant breeders today has the potential to increase the gain of selection. However, they also result in numerous complex choices in the design of efficient crossing and selection strategies. Computer simulations are essential to optimize breeding programs that are multi-year, high-effort endeavors and to compare process efficiencies without going through field experiments, thus saving both time and field resources. In addition, computer simulations are key to evaluate statistical properties of new methods and procedures as well as to exploit crop growth models and G*E interactions. The first two areas are discussed in great detail in our review. Furthermore, the review evaluates the capabilities, assumptions, and limitations of all publicly available simulation tools highlighting their relevance for the different areas of application.

## Introduction

Plant breeding has played (Fehr 1987), and continues to play (Xiong et al. 2022), a pivotal role in shaping agricultural landscapes and ensuring food security worldwide. The origins of plant breeding can be traced back to ancient civilizations, where farmers selectively cultivated crops to improve yield, adaptability, and nutritional quality (Harlan 1992). Traditional breeding methods, such as mass selection, controlled crossing, and recurrent selection, laid the groundwork for crop domestication and varietal improvement (Doebley et al. 2006). In the 19th century, a new era of systematic breeding practices started, leading to the development of hybridization techniques and the emergence of crop improvement programs worldwide. However, the revolution of molecular genetics, biotechnology, and computational sciences in the last decades has transformed the field of plant breeding enabling breeders to directly measure and manipulate the genetic code (Cooper et al. 2002). The now routinely applied marker-assisted selection (MAS), genomic selection (GS), and genome editing techniques enable precise modification of target genes and are unprecedented tools and methodologies (Kumar et al. 2024) which accelerate the breeding progress. Moreover, high-throughput phenotyping technologies facilitate rapid trait evaluation, enhancing breeding efficiency and, consequently, the genetic gain (Fiorani and Schurr 2013). Altogether, these tools and methods offer breeders unprecedented opportunities to accelerate the development of high-yielding, stress-tolerant cultivars tailored to the needs of farmers and consumers in a rapidly changing world.

## Relevance of computer simulations for plant breeding

The wide range of tools and methods available to plant breeders today also results, however, in complex choices in the design of efficient crossing and selection strategies (e.g. Yoosefzadeh-Najafabadi 2025). Quantitative genetics provides much of the framework for informing these decisions and, thus, supports the design and analysis of selection methods used within breeding programs (Falconer 1996; Lynch 1998). However, the basic assumption underlying the formulation of the classical quantitative-genetic theory is the polygenic and in many cases purely additive nature of the variation (Falconer 1996). It is widely recognized that these assumptions were made to enable tractable treatments of the quantitative-genetic models (Cooper et al. 2002). Model developers widely recognize that these frameworks are approximations of the genetic architecture of quantitative traits (Cooper et al. 2002). Some of these assumptions can be easily tested or satisfied by experiments; others can seldom, if ever, be met (Wang and Pfeiffer 2007).

**Fig. 1:**
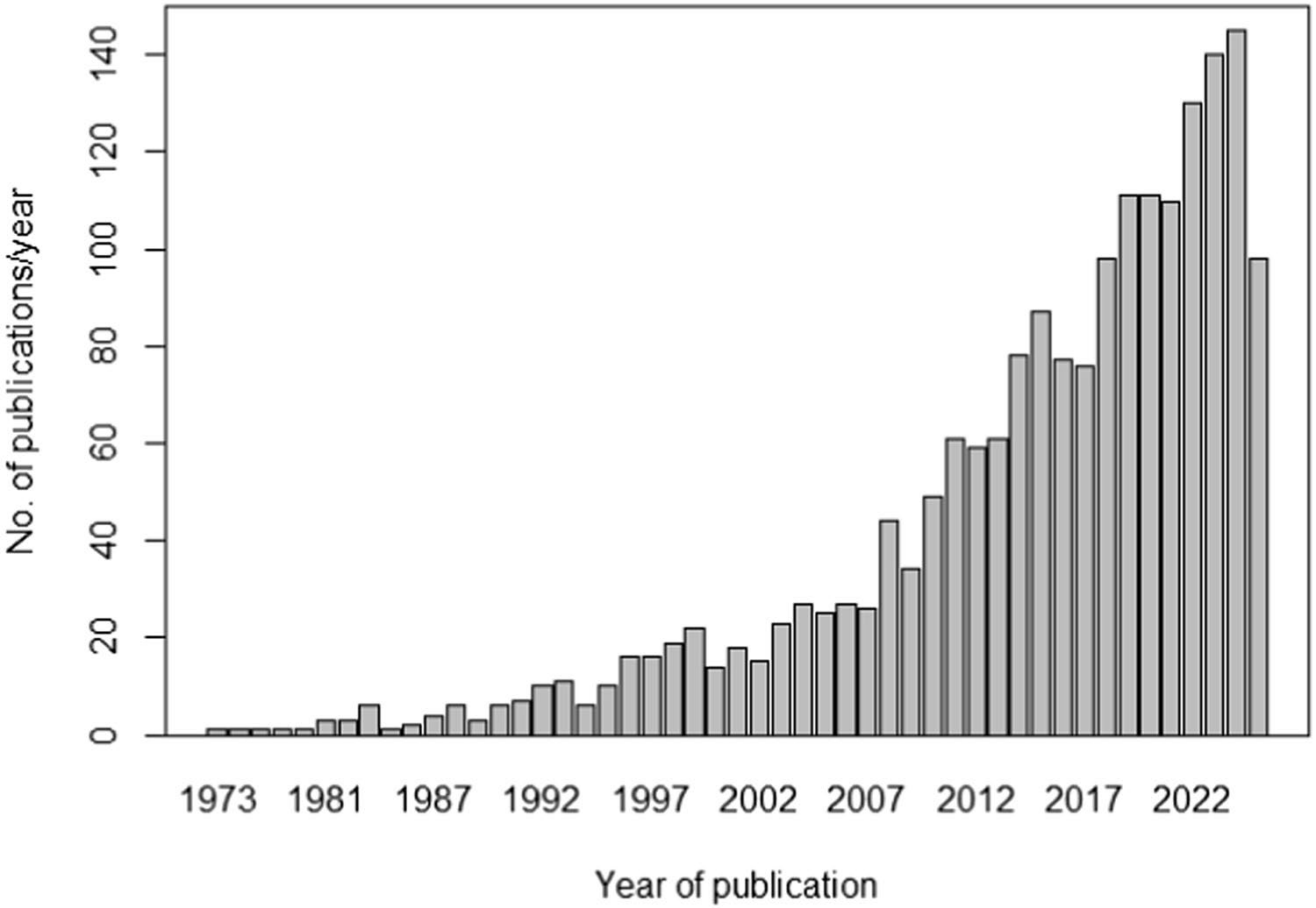
Annual number of publications containing ’Simulations’ AND ’Plant Breeding’ in their full text (extracted from Web of Science, July 14, 2025)

Computer simulations allow us to investigate the implications of relaxing some of the assumptions and the effect this has on the conduct of a breeding program without the need for extensive experimental work. Therefore, computer simulations can be used to gain an understanding of the relationship between the efficiency of a breeding strategy and the genetic architecture of traits (Cooper et al. 2002). Furthermore, analytical approaches are challenging and in some cases even impossible to derive for complex cross and selection programs, such as parental selection, pedigree relationships, linkage, and recombination whereas simulation experiments can easily accommodate all of these factors. Therefore, computer simulations are particularly key to optimize breeding programs that are multi-year, high-effort endeavors and to compare process efficiencies without going through field experimentation, thus saving both time and field resources (Wang et al. 2003). This explains why computer simulations have been increasingly used in quantitative genetics to support decision making in the design of plant breeding programs (Fig. 1).

The following earlier manuscripts summarized the status of computer simulations in plant breeding in form of review articles (Fraser and Burnell 1970; Cooper et al. 2002; Wang and Pfeiffer 2007; Li et al. 2012). Because of the above-mentioned increasing use of computer simulations in plant breeding but also the high number of software tools that have recently been developed and published, we considered it important to summarize the features, functionalities, and underlying assumptions of currently available software tools.

## Areas of application of computer simulations

The most important and also most frequently reported applications of computer simulations in a plant breeding context can be grouped into three different areas of application: (i) breeding methodological considerations, (ii) evaluation of statistical properties of new methods and procedures, and (iii) exploitation of crop growth models and G*E interactions. These areas are discussed in greater detail below. Due to space constraints and the vast body of literature in this field, we regret that we could not cite all relevant primary studies. Instead, we have selected representative works that best illustrate the key concepts of the areas of application.

### Breeding methodological considerations

The complexity of breeding programs offers, on one hand, the flexibility to design freely but, on the other hand, requires many decisions each with multiple alternative solutions. In most situations, extensive experimental evaluation of breeding programs is impossible or at least beyond the resources typically available in individual breeding programs. Therefore, simulation tools are important in designing and testing alternative breeding strategies.

Breeding programs can be classified according to many alternative criteria. We decided to separate in this review article two types of breeding programs when summarizing the use and result of computer simulations: (i) breeding programs aiming to transfer major effect genes or quantitative trait loci (QTL) which are hereafter referred to as introgression programs and (ii) those exploiting small effect genes or QTL.

#### Optimization of introgression programs

When one or more major genes underlying the trait of interest are known, these can be introgressed in a genotype of interest using backcrossing methods. The application of molecular markers in this context is designated as marker-assisted backcrossing (MABC) (e.g., Tanksley 1983). This method employs molecular markers to identify and select for target genes during the backcrossing process (i.e., foreground selection). At the same time, this selection is ideally coupled with assessing the degree of recovery of the genome of the recurrent parent (i.e., background selection). Over the years, significant progress has been made in understanding the principles and applications of MABC. In the context of this review article, we focused on summarizing the major outcomes of a selected set of computer simulation studies.

The design of MABC programs was studied with respect to the introgression of single dominant and recessive genes (Hospital et al. 1992; Frisch et al. 1999a, b; Frisch and Melchinger 2001a), two (Frisch and Melchinger 2001b), or multiple genes (Servin et al. 2004). Most simulation studies focused on the selection of foreground and background chromosome segments under different marker densities and number of generations needed to recover the recurrent parent’s genome. In general, background selection proved more effective when applied after foreground selection (Hospital and Charcosset 1997). Frisch et al. (1999a) e.g., demonstrated through computer simulations that increasing population sizes from generation BC1 to BC3 can reduce the number of required marker data points by up to 50% while keeping the recurrent parent genome at the same level. The simulations of Frisch and Melchinger (2001b), which focused on the introgression of two genes, suggested that gene enrichment during early generations can greatly reduce resource requirements.

As many of the agronomically important traits are quantitative traits (Bernardo 2002) and, thus, are influenced by many genes, several studies considered the backcrossing of QTL (Hospital and Charcosset 1997). The simulations by Hospital and Charcosset (1997) indicated that three markers, one at the center of a QTL confidence interval (CI) and two flanking markers, are necessary to confirm the existence of a target gene after several backcross generations. However, for less precisely mapped QTL, introgressing a whole segment containing the target gene may be necessary, albeit with the risk of introducing more deleterious genes into elite lines, thereby decreasing selection efficiency. Finally, two different approaches for multi-QTL introgression were compared (Hospital and Charcosset 1997): simultaneous design (introgress several QTL at the same time) and pyramidal design (introgress a single QTL at the first stage and then combine into a single genotype). Simulations showed that to introgress four QTL simultaneously, 1600 individuals are necessary to obtain a target genotype with 99% confidence. In contrast, with a pyramidal design, the total number of individuals required would be 580. More recently, MABC for developing libraries of near-isogenic lines was studied (Peleman and van der Voort 2003; Falke et al. 2009).

All previously mentioned studies in the context of introgression programs focused on optimizing the number of genotyped individuals as well as the positions and density of background selection markers with respect to the required number of marker data points. However, high-throughput marker systems based on single nucleotide polymorphisms (SNP) have been developed for many crops. Due to the high level of automation of these systems such as SNP arrays, they allow for cheap and fast analysis of hundreds or thousands of marker loci in a single analysis step (Gupta et al. 2001; Syvänen 2005). The exploitation of such genotyping systems was studied with the help of computer simulations by Herzog and Frisch (2011). Their results suggested that a three-stage selection strategy that combines selection for recombinants at markers flanking the target gene with single marker assays and genome-wide background selection with high-throughput markers in the first backcross generation was more efficient than genome-wide background selection with high-throughput markers alone.

#### Optimization of breeding programs targeting quantitative traits

Although the introgression of major genes is essential for the success of breeding programs in many crops, for most traits of agronomic importance no major genes have been described (Bernardo 2002). Therefore, alternative ways of manipulating such traits have to be applied and this is performed in classical breeding programs. A breeding program is a complex enterprise in the sense that it is based on multiple steps where each step allows multiple alternatives. Limited financial resources but also operational aspects constrain the design of breeding programs. Therefore, the optimization of breeding programs i.e., the combination of all these decisions to obtain the best possible outcome under the given circumstances is a highly complex but also a context dependent procedure. Thus, every time new methods or approaches become available, their optimal implementation needs to be evaluated—in many cases for every crop, as the context e.g., variance components or cost scenarios are different.

In the following, some example studies that reported such optimizations are given. With the advent of efficient double haploid (DH) creation methods in maize, Longin et al. (2006) and Wegenast et al. (2008) examined the efficient implementation of this method. Their computer simulations suggested that with further improvements in the DH technique and the realization of more than two generations per year, early testing of S(1) families prior to the production of DH lines would become very attractive in hybrid maize breeding. McClosky and Tanksley (2013) examined through a series of computer simulations whether increasing recombination beyond normal levels will result in significant gains in short-term selection.

A considerable number of studies focused on the implementation of marker-assisted or genomic selection. Fewer studies evaluated the potential of integrating data from high-throughput phenotyping in the breeding process. In the manuscript of Kuchel et al. (2005), computer simulations were used to design a genetically effective and economically efficient marker-assisted breeding strategy for wheat integrating both restricted backcrossing and DH technology. Lorenz (2013) studied using computer simulation the resource allocation on response to MAS and genomic selection in a single biparental population of DH lines.

Longin et al. (2015) optimized the allocation of resources in different breeding strategies by predicting the expected selection gain for a fixed budget. In this study, classical two-stage phenotypic selection was compared with three genomic selection based breeding strategies for line and hybrid breeding in wheat. The study of Gorjanc et al. (2018) evaluated optimal cross selection to balance selection and maintenance of genetic diversity in two-part plant breeding programs with rapid recurrent genomic selection. Muleta and Pressoir (2019) used simulations to identify conditions under which genomic-assisted recurrent selection would be more effective than phenotypic recurrent selection in a young breeding program of sorghum in a developing country. Phenotypic recurrent, marker-assisted recurrent, and genomic selections for both short- and long-term breeding procedures were compared by Ali et al. (2020). The optimal integration of genomic selection in clone breeding programs of tetraploid species maximizing the short-term and long-term gain of selection was studied by Wu et al. (2023) and Wu et al. (2025), respectively. Galli et al. (2021) made inferences regarding the potential of plant high-throughput phenotyping for breeding programs via computer simulations.

### Evaluation of statistical properties of new methods and procedures

In the context of plant and animal genetics and breeding, various methods and approaches for analyzing experimental data are developed. This typically requires the benchmarking of the newly developed vs. the established methods and approaches which is done in many cases using computer simulations. For example, Stich et al. (2007) examined the power to detect higher-order epistatic interactions in a metabolic pathway using the nested association mapping strategy. Li (2011) proposed a two-stage procedure for multi-SNP modeling and analysis in genome-wide association mapping (GWAS) by first producing a ‘preconditioned’ response variable using a supervised principal component analysis and then formulating Bayesian lasso to select a subset of significant SNP. The performance of this method was evaluated based on computer simulations. A new method for GWAS called fixed and random model circulating probability unification (FarmCPU) was proposed by Liu et al. (2016). Both real and simulated data were analyzed and demonstrated that FarmCPU improves statistical power compared to current methods.

Furthermore, computer simulations were essential to characterize and validate genomic prediction models. Daetwyler et al. (2013) e.g., reviewed simulation procedures, discussed validation of results, and benchmarked a variety of genomic prediction methods using simulated and experimental data. An overview of available methods for implementing parametric genomic prediction models as well as the lessons learned from simulation and empirical data analysis was provided by de Los et al. (2013). More recently, machine learning methods are becoming widely advocated for and used in genomic prediction studies. Lourenço et al. (2024) e.g., comparatively evaluated the genomic predictive performance of several groups of supervised machine learning methods, specifically, regularized regression methods, deep, ensemble, and instance-based learning algorithms.

Also for the evaluation of SNP and structural variant detection procedures, computer simulations are frequently applied. The study of Schilbert et al. (2020) provided a comparison of the performance of the most frequently applied tools for SNP and InDel detection in plant genomes. Yang (2020), Weisweiler et al. (2022), Weisweiler and Stich (2023) e.g., used computer simulations to benchmark various tools to detect structural variants based on short reads in the pear, barley, and potato genome, respectively.

### Crop growth models and G*E interactions

Crop growth models are valuable tools in plant breeding. These models allow the prediction of plant performance under various environmental and management conditions and therewith enable breeders to make informed decisions without extensive field testing. Furthermore, crop growth models, especially those that integrate physiological characters, allow predicting how specific traits contribute to yield and adaptation. Therewith, such models assist in defining ideotypes i.e., the ideal plant types suited for future climate scenarios and farming systems. A detailed review of crop growth models has been provided by Rötter et al. (2015) and is therefore not provided in our review.

Integrating genomic prediction with crop growth models has become a focal point in agricultural research, aiming to enhance the accuracy of predicting plant performance across diverse environments (Onogi 2022). Technow et al. (2015) provided a proof of concept study which illustrated that approximate Bayesian computation allows the incorporation of a crop growth model directly into the estimation of whole genome marker effects in genomic prediction models. These authors showed that this approach can be considerably more accurate than the benchmark GBLUP model in predicting performance in environments represented in the estimation set as well as in previously unobserved environments for traits determined by non-additive gene effects. This approach was later designated as genomic prediction-assisted crop growth models, in which genomic prediction is used to predict parameters of crop growth models for new genotypes. Several studies applied this approach to e.g., data sets of rice, maize, cauliflower, and wheat (Onogi et al. 2016; Cooper et al. 2016; Rosen et al. 2018; Jighly et al. 2023).

An alternative to genomic prediction-assisted crop growth models is crop growth models-assisted genomic prediction, where phenotypes of new genotypes are predicted with genomic prediction. Largely three approaches have been proposed which have been described in detail by Onogi (2022) and, thus, are not presented here in detail. Note that genomic prediction-assisted crop growth models and crop growth model-assisted genomic prediction will have different roles in plant breeding. Although both methods predict phenotypes, the latter predicts breeding values and, thus, this method is suitable for selecting candidates to increase genetic gain. In contrast, a genomic prediction-assisted crop growth model is suitable for designing ideal phenotypes under real or simulated environmental conditions.

## Basic steps in designing simulation studies in a plant breeding context

In the first step, the objectives of the simulations must be defined, i.e., the dependent variables of interest will be compared among the different simulation scenarios based on the variation of which parameters? Upon the information about the assumptions and built-in parameters of the simulation tools, one or several of those are selected for the actual simulations. However, before the simulations are executed, the realization of the parameters are selected based on own or published experimental data or based on theoretical considerations. In the next steps, the actual simulations are run and the simulation data are analyzed to derive the realizations of the dependent variable of interest. The results are then evaluated for their plausibility and potentially also compared to experimental data. In the final step, conclusions are drawn. These basic steps are in good agreement with the steps that are also executed when planning computer simulations in other research areas e.g., evolutionary genetics Hoban et al. (2012).

In order to make this explanation of the basic steps of computer simulations more concrete, it will be illustrated based on the simulation study of Wu et al. (2023) that was mentioned above. The objective of this study is to assess as dependent variable the expected selection gain for a clone breeding program of potato. The parameter that is altered in this study is the considered breeding strategy. In detail, the authors compared a phenotypic selection scheme with different genomic selection selection schemes. As mentioned above, the next step is the selection of the realization of the parameters based on own or published experimental studies or based on theoretical considerations. In the example of Wu et al. (2023), the authors chose as benchmark the dimensioning and cost scenario from a classical clone breeding program of potato. This was compared with respect to its genetic gain to several breeding schemes involving genomic selection, where the total cost of each scheme was identical to that of the standard scheme. The genetic variance components and prediction accuracies that were the basis for the simulations were taken from experimental studies (e.g. Thelen et al. 2025, 2025a). AlphaSimR (Gaynor and Cgorjanc 2021) was used for this simulation study. The results of the simulation were then compared to those of earlier simulation studies and model calculations to evaluate their plausibility. The results illustrated that implementing genomic selection in consecutive selection stages can largely enhance short-term genetic gain and that it is recommended to implement genomic selection at the earliest at the single hill or A clone stage. Furthermore, it was observed for selection strategies involving genomic selection that the optimal allocation of resources maximizing the genetic gain of the target trait differed considerably from those typically used in potato breeding programs and, thus, require the adjustment of the selection and phenotyping intensities.

## Tools for computer simulations in plant breeding

A wide range of software tools has been developed for computer simulations in a plant breeding and genetics context. Several of these tools such as Gregor (Tinker and Mather 1993), Plabsim (Frisch and Bohn 2000), Plabsoft (Maurer et al. 2008), MBP (Gordillo 2008), and AlphaSim (Faux et al. 2016) have been frequently used in the past (Sun et al. 2011) but, to the best of our knowledge, are no longer available. Therefore, these will not be presented in our review.

For those tools that are still publicly available, we did our best to collect key facts as well as to provide information about the models and assumptions underlying each tool as well as the basic functionalities (Table 1). However, due to the diversity in the functionality of these programs, we are not able to provide a head-to-head comparison of program efficiency and outcome.

The available simulation tools can be categorized according to various aspects. To enable readers to quickly identify the relevant tools, we ordered them based on the area of application which were outlined in the previous chapter. In the following, we provided detailed information for each of these tools starting with the ones that are suitable for using them for breeding methodological considerations.

### Tools suitable for breeding methodological considerations and evaluation of statistical properties of new methods

#### QU-GENE and its application modules

QU-GENE (Podlich and Cooper 1998) is the oldest simulation platform that is still available. It was developed for simulations of diploid species and can consider multiple alleles, additive, dominance, as well as epistatic effects. Recombination is simulated as described by Fraser and Burnell (1970), but recombination interference is not considered. QU-GENE relies on the core E(N:K) genetic model, where E is the number of types of environment, N is the number of genes, K indicates the level of epistasis and the parentheses indicate that different N:K genetic models can be nested within different environments. This model deviates from what is used by other simulation tools described below. QU-GENE allows the simulation of G*E interactions. However, QU-GENE was originally designed without considering more complicated genetic effects and/or genetic phenomena, such as mutations, cytoplasm effects, and fertilities of female and male gametes, including interactions between cytoplasmic and nuclear genes.

Simulations with QU-GENE follow two stages. In the first step, QU-GENE serves as the engine and, thus, all the genetic and environmental information for the simulation experiment are defined in it. Further, the base germplasm and/or a reference population to estimate genetic and/or environmental variances is generated. In the second step, one of the specific application modules such as QuLine (Wang and Dieters 2008), QuHybrid (Wang 2008a), QuMARS (Li 2011), or QuLinePlus (Hoyos-Villegas et al. 2019), which have been developed over time and are suitable for various breeding situations, are used.

QuLine is the breeding module that is used for simulating self-pollinating species. It allows to model breeding strategies in classical breeding, but also allows the investigation of the use of newer technologies, such as DH or MAS. The QuLinePlus breeding module is an extension of QuLine and is used for cross pollinated species with half-sib mating strategies. The QuHybrid breeding module can be used for simulating hybrid breeding programs incl. test cross situations. QuMARS is the breeding module that is used for simulating recurrent selection strategies, and is used to optimize the integration of phenotypic selection, marker-assisted recurrent selection, and genomic selection in breeding programs.

#### QMSim

QMSim (Sargolzaei and Schenkel 2009) is a software tool that was developed in the context of animal breeding to simulate large-scale genotyping data sets in multiple and complex livestock pedigrees. Therefore, it can only consider diploid species with additive effects following different distributions. QMSim simulates meiosis using a Poisson model with the possibility of considering crossover interference. Simulations with QMSim follow two main steps. In the first step, a historical population is simulated to create mutation-drift equilibrium and initial linkage disequilibrium. In the second step, recent population structures are simulated. QMSim was intensively used to validate new approaches for the use of genomic selection as well as new approaches for fine mapping but it also allows the selection of individuals based on phenotypes, estimated, and true breeding values.

#### Breeding scheme language (BSL)

BSL (Yabe et al. 2017) is a simulation platform for breeders that was developed to flexibly evaluate the performance of different breeding schemes. This tool, which was designed for diploid species, is able to simulate additive, dominance, and epistatic effects but no G*E interactions. The founder population is simulated using the coalescent-based whole genome simulator GENOME (Liang et al. 2007), where experimental haplotype data can serve as input for the founder. BSL allows crossing, selfing, and DH production as mating types. A selection based on phenotypes, genomic estimated breeding values, or randomly is possible.

#### ADAM-plant and ADAM-multi

ADAM-plant is a software tool that models breeding schemes for self- and cross-pollinated crop plants using stochastic simulations (Liu et al. 2019). It was developed based on the software ADAM, which was suited to simulate animal breeding programs (Pedersen et al. 2009). The founder population that is needed for the simulations is in ADAM-plant either generated as a recombination-drift-mutation-selection equilibrium population by using a Fisher-Wright inheritance model or can be imported e.g., from experimental genomes. ADAM-plant allows the simulation of different breeding schemes including genomic selection, speed breeding, and overlapping breeding cycles. Specifically, with ADAM-plant the mating types cloning, crossing, selfing, and DH production can be simulated.

ADAM-plant simulates meiosis. The number of crossovers is drawn from a Poisson distribution with their mean number equal to the length of the chromosomes in Morgan. The crossovers are then placed randomly along the chromosome. Crossover interference is not considered by ADAM-plant.

The genotypic values of the diploid genotypes can be calculated from an additive effect model. ADAM-plant also allows the consideration of G*E interactions. Multiple traits can be simulated that are, if necessary, correlated by shared QTL.

The selection process in ADAM-plant can be carried out on single or multiple traits by truncation selection based on various criteria (phenotype, (genomic) best linear unbiased predictors, or optimum contribution selection). ADAM-plant offers the possibility to execute selection within a family or across families.

ADAM-multi described by Chu and Jensen (2025) is an extended software of ADAM and ADAM-plant to simulate complex breeding programs for animals and plants. In contrast to ADAM and ADAM-plant, it can be used for polyploid species. The recombination events follow bivalent chromosome pairing (Voorrips and Maliepaard 2012), implying that double reduction is not considered. The genetic model of ADAM-multi can consider not only additive effects but also dominance and epistatic effects as well as multi-allelic effects for each QTL and therewith increases very much the possibilities of ADAM-plant, which only considers additive effects and bi-allelic effects.

#### Modular breeding program simulator (MoBPS) and MoBPSweb

Another tool that allows to simulate complex breeding programs for diploid organisms is MoBPS. It was developed also for an application in animal breeding and therefore it allows the specification of sex chromosomes. The founder genotypes for the simulation can be either simulated by random mating for a high number of generations or imported e.g., from experimental data. MoBPS allows selfing, DH, clonal propagation, designated crossing, and genome editing as mating types where meiosis is considered. Recombination events are assumed to follow a Poisson distribution with one expected recombination per one Morgan. Alternatively, a function for the sampling of recombination events can be provided.

The genotypic values of the diploid genotypes can be calculated by MoBPS from additive, dominance, and epistatic effects. MoBPS also allows the consideration of G*E interactions. Multiple traits can be simulated that are, if necessary, correlated. Selection in MoBPS is executed on estimated breeding values from different external models/software, phenotypes, or selection indices for multiple traits. Even though basically all information regarding each individual is stored, Pook and Schlather (2020) emphasized that the required memory in MoBPS is still relatively low as a highly efficient storage structure is used.

With MoBPSweb, Pook et al. (2021) described a web-based version of MoBPS which allows to run the simulations on a virtual machine which can be accessed via an internet browser.

#### AlphaSimR

AlphaSimR described by Gaynor and Cgorjanc (2021) is the follow-up tool of AlphaSim (Faux et al. 2016) which was one of the most frequently used simulation tools of the last years. AlphaSimR is an R package for stochastic simulations of entire plant and animal breeding programs. The founder haplotypes are simulated using Markovian Coalescent Simulator (Chen et al. 2009). Alternatively, experimental or external haplotypes can be used as input as well. AlphaSimR allows inbred and outbred systems, crossing, DH, and clonal propagation as mating types.

For diploid species, genetic recombination is modeled based on gamma model (McPeek and Speed 1995), which allows crossover interference, where the magnitude of interference can be adjusted. For autopolyploid species, AlphaSimR considers bivalent and quadrivalent chromosome pairing. Bivalent pairing follows the scheme of diploid species and quadrivalent pairing follows the model used in the PedigreeSim software (Voorrips and Maliepaard 2012).

Genotypic values can be calculated in AlphaSimR from additive, dominance, and epistatic effects. Dominance effects in autopolyploid species are modeled in digenic form and the epistatic effects are modeled as additive by additive interactions effects between discrete pairs of loci. AlphaSimR also allows the consideration of G*E interactions, where these effects are modeled as additive effects whose value is a function of a single environmental covariate. It is possible to use phenotypes, genetic values, breeding values, estimated breeding values, user’s criteria for a single trait, or an index of multiple traits for selection. The selection can be executed as selection between or within families, or across an entire population.

#### Blib

Blib (Zhang et al. 2022) was developed to offer a simulation tool that offers besides the comprehensive genetic models of QU-GENE also the possibility to consider more complicated effects and/or genetic phenomena, such as mutations, cytoplasm effects, fertilities of female and male gametes, and interactions between cytoplasm and nuclear genes. Blib, which was developed for diploid species, comprises currently three specific modules. The first one is called DRIFT and was developed to simulate genetic drift in random mating populations. The second Blib application module called PRS was developed to simulate phenotype based recurrent selection. The third application module, called ISB, was developed to simulate pure line, hybrid, and clone breeding programs and simulates genetic recombination events between parents with designed crossing schemes. Li et al. (2025) described in great detail the applications of ISB.

#### ChromaX

ChromaX is a Python library that enables the simulation of genetic recombination, the calculation of genomic estimated breeding values, and selection processes of diploid species (Younis et al. 2023). In contrast to the above-described simulation tools, ChromaX does not offer the possibility to simulate a founder population. Instead, phased genetic data of the populations are required as input data from external sources. Another limitation of ChromaX is that in contrast to additive and G*E interactions effects (Faux et al. 2016) dominance and epistatic effects cannot be considered for the calculation of the genotypic values. Genetic recombination is simulated by ChromaX and a Poisson model for crossover interference is assumed (McPeek and Speed 1995).

ChromaX was designed for the simulation of breeding programs of self- or open-pollinated species. Other systems, such as hybrid breeding from distant heterotic groups or monoecious/dioecious reproductive systems are not considered. The executed selection can be based on breeding values, phenotype, or optimal haploid value (Daetwyler et al. 2015).

The functions of ChromaX are compiled in XLA (Accelerated Linear Algebra) (Sabne 2020) which allows ChromaX to run seamlessly on various devices exploiting the parallelization offered by a variety of high-performance computing devices. The computational efficiency of ChromaX was benchmarked vs AlphaSimR and was considerably higher (Younis et al. 2023).

#### SNPcan breeder

SNPcan breeder is a software that enables the simulation of breeding programs using simulated individual whole genome data (Degen and Müller 2023). SNPcan breeder was developed in the context of tree breeding. Compared to many of the above-described tools it offers less flexibility first with respect to the underlying genetic architecture but also to the considered breeding schemes. SNPcan breeder assumes diploid sets of chromosomes and co-sexual individuals (monoecious or hermaphroditic). The genotypic values are modeled on the basis of additive effects. In addition, inbreeding depression effects are considered by modeling a linear reduction of genotypic values in dependence of the degree of homozygosity. SNPcan breeder does not allow the consideration of G*E interactions.

For the simulation of genotypic values, the number of causal SNP is specified and one of three alternative functions for the distribution of the allelic effects is selected: (a) negative exponential distribution, (b) normal distribution, or (c) uniform distribution. The considered SNP can be bi-, tri-, or tetra-allelic, where their proportions as well as the distribution of allele frequencies can be defined. In the next step, the SNP are equally distributed across all chromosomes, where the chromosomes are assumed to have equal sizes. The probability for a crossover is identical along the chromosomes.

SNPcan breeder allows the simulation of the following mating designs: diallel, half-diallel, disconnected half-diallel, factorials, and random mating. Furthermore, this tool enables to execute selection based on phenotypes, breeding values from progeny tests, MAS based on GWAS results, and genomic selection.

#### Python breeding optimizer and simulator (PyBrOpS)

PyBrOpS is a Python package capable of simulating breeding pipelines (Shrote 2024). It provides a framework for customization and expansion, allowing users to integrate new functionalities or extend existing ones as their research goals or breeding programs evolve.

The founder population but also the genetic map information can be randomly simulated or imported from external tools or experimental data. PyBrOpS allows to calculate genotypic values based on additive and dominance effects. Epistatic and G*E interaction effects are not considered, but they may be included by custom models. Meiotic recombination is modeled by PyBrOpS. In this context, two genetic map functions, namely Haldane’s (Haldane 1919) or Kosambi’s (Kosambi 1943) map functions, can be considered. The manual describes that various ploidy levels can be simulated. However, the way of codification of additive and dominance effects implies a diploid inheritance. In addition, the process of simulating meiosis does not consider the special aspects of autopolyploid species.

PyBrOpS allows to execute single- or multi-trait selection based on phenotypes, genomic estimated breeding values, optimal haploid values, optimal population values, expected maximum breeding values, and weighted genomic breeding values. The implemented mating types are selfings, two-, three-, and four-way crosses, as well as DH production.

Among the above-described tools, which allow the simulation of entire breeding programs, QU-GENE and its offsprings, QMSim, as well as Alphasim and its offspring have been so far most frequently used, but one has to say that these are also among the ones that have been described first. Further tools exist which are not designed to model entire breeding programs with their complex steps but are designed to either model individual steps of breeding programs or to establish data with which novel statistical methods can be benchmarked. These are categorized in Table 1 as being suitable for the evaluation of statistical properties of new methods.

### Tools suitable for the evaluation of statistical properties of new methods

#### QuantiNemo and QuantiNemo 2

QuantiNemo (Neuenschwander et al. 2008) was developed to investigate the effects of selection, mutation, recombination, and drift on one or multiple quantitative traits with varying architectures in structured populations. Quantitative traits are simulated for diploid species based on additive, dominance, and epistatic effects. Several mating systems are available in QuantiNemo: random mating or selfing for hermaphrodites, promiscuity, monogamy or polygyny for species with separate sexes. QuantiNemo allows to execute stabilizing and directional selection, where the strength and direction of selection may vary for each trait and population. Furthermore, the selective pressures can change over time. In their review, Hoban et al. (2012) acknowledged that QuantiNemo was the software package for evolutionary genetic simulations with the highest degree of flexibility.

The tool QuantiNemo 2 which was described in 2019 (Neuenschwander et al. 2019) offers even more flexibility. Compared to QuantiNemo, QuantiNemo 2 displays among others the following new features:

- the possibility to switch from individual-based to population-based simulation with coalescence,
- the sex of an individual to be determined genetically,
- the possibility to simulate fitness landscapes of any shape and, thus, any number of optima,
- an improved flexibility of use,
- cloning as a new mating system,
- new population growth models,
- corrected and more realistic density-dependent dispersal rates,
- the genetic map can now evolve with time and the distance between loci can accommodate quantitative traits using recombination factors.

#### MaCS

MaCS (Chen et al. 2009) is a computer simulation tool that simulates the DNA sequence of a founder population using Markovian coalescent simulations. In this function it is used by other tools such as AlphaSimR. MaCS is fully flexible with regard to historical population size, mutation and recombination rates, genome size, and number of founders generated. As it was developed in the context of human genetics, it was designed for diploid genomes. A simulation of geno- or phenotypic values by MaCS is not possible.

#### PedigreeSim

Another tool that is capable of simulating genotypic information but no phenotypic values of individuals is PedigreeSim (Voorrips and Maliepaard 2012). The unique aspect of PedigreeSim at the time of publication was the possibility to simulate the genotypes in diploid and autopolyploid species. For diploids, crossover interference can be assumed or not. For autopolyploids, a variety of models can be used, including both bivalent and quadrivalent formation, varying degrees of preferential pairing of homologous chromosomes, or different quadrivalent configurations. In addition, varying probabilities of preferential versus random pairing of chromosomes can be simulated, allowing to simulate allo- as well as autopolyploids and intermediate forms. These models are used by other simulation tools such as AlphaSimR (Gaynor and Cgorjanc 2021), if quadrivalent pairs are considered.

#### PopVar

PopVar (Mohammadi et al. 2015) is a R package that allows to simulate progeny for crosses among diploid genotypes to predict the population mean and variance in the situation of an additive inheritance. Furthermore, it also performs cross-validation to estimate genome-wide prediction accuracy of multiple statistical models.

#### XSim and XSim version 2 (XSimV2)

XSim developed by Cheng and Garrick (2015) is another tool that focused on the simulation of genotypes by simulating sequence data in descendants of various pedigrees. The founder of sequence can be simulated or experimental data can be considered. XSim allows to define allele frequencies, map positions, number of loci, chromosome lengths, number of chromosomes, and mutation rates. Crossover interference is considered during the simulations.

In 2022, a second version of XSim, named XSimV2, was described (Chen et al. 2022) which now allows the possibility to simulate phenotypes. XSimV2 simulates founders using a Bernoulli distribution with the event probability equaling to 1—minor allele frequency. The considered mating schemes are random mating, diallel crosses, and selfing. In addition, DH production in plants and embryo transfer in animals are available as mating types. The genetic map position is used to simulate crossover events. Phenotypic values are calculated from additive effects only where only diploid and allopolyploid species are considered. XSimV2 allows to execute selection based on phenotypes, estimated breeding values, as well as a selection index for multiple traits. Therewith XSimV2 belongs to the category of tools that are suitable for breeding methodological considerations.

#### Sequence-based virtual breeding (SBVB) and polyploid sequence based virtual breeding (pSBVB)

The software tool SBVB described by Pérez-Enciso et al. (2017) uses real sequence data with the specified genetic architectures and pedigree to simulate genomes and phenotypes of new offspring. In order to do so, phased genomes for founders are required. It is possible to specify a recombination map, genetic architectures of traits, and pedigree, etc.. SBVB assumes by default a cM to Mb ratio of one which alternatively can be specified by the user. By default, the program generates up to three crossovers per chromosome per generation. The actual number is sampled from a truncated Poisson distribution. No crossover interference is considered during the simulation of meiosis. SBVB allows the simulation of the effect of sex chromosomes. This tool, which was developed for diploid species, is able to simulate additive, dominance, and epistatic effects but no G*E interactions.

Zingaretti and Monfort (2019) described with pSBVB a modification of SBVB that is suitable to simulate genomes and phenotypes of polyploid species. As phased genomes are rarely available for polyploid genomes so far, pSBVB randomly generates a phase configuration in its simulations, if the phase is not provided. The specificities of polyploid meioses are considered by pSBVB. In allopolyploids, disomic inheritance, resulting from preferential pairing between homologous chromosomes is assumed. In contrast, in autopolyploids, the probability of recombination between homologs can be specified via a recombination matrix. With respect to the simulation of quantitative phenotypes, the difference between SBVB and pSBVB is that the latter does not allow the simulation of epistatic effects in contrast to the previous tool.

#### *GeneEvolve*

GeneEvolve allows to simulate individual-level genome-wide data for diploid species based on a reference panel of phased chromosomes (Tahmasbi and Keller 2017). In addition, phenotypic values and selection events can be simulated. However, it is difficult to imagine how all steps of a breeding program can be modeled by GeneEvolve.

Recombination rates between loci can be estimated from previously simulated genotype data or, for more realistic simulations, taken from real, phased SNP or sequence datasets, or published recombination maps. GeneEvolve simulates phenotypic values based on additive and dominance effects only within families and does not allow to consider epistatic effects. In addition, unique, familial, shared sibling, and population specific environmental values can be considered. For multiple phenotypes, the phenotype is a user-specified linear combination of each phenotype. The user can also choose monogamous or polygamous mating systems and can disallow close inbreeding.

#### *SeqBreed*

SeqBreed described by Pérez-Enciso et al. (2020) is a software tool to simulate populations under (genomic) selection which inherits most of its functionality from SBVB and pSBVB. It can be used to simulate genomic selection experiments, study the performance of GWAS, etc., but it is not designed to model entire breeding programs. Founder sequence genotypes are loaded from vcf or plink format. Offspring genomes and phenotypes can be simulated under selection or random drift. Recombination rates can be specified, where the sex chromosomes are assumed to be non-recombining and for the mitochondrial chromosome, which is also considered a non-recombining chromosome, a maternal inheritance is assumed. SeqBreed allows to consider the specificities of autopolyploid meiosis with the exception of double reduction.

Desired heritabilities as well as causal SNP and their effects for every trait can be specified flexibly in SeqBreed. When doing so, additive and dominance effects can be modeled. In contrast, epistatic as well as G*E interactions cannot be considered in the simulations using SeqBreed.

#### *FieldSimR*

Compared to above-mentioned simulation tools, FieldSimR serves a different purpose. Instead of simulating genotypes and their phenotypes, it simulates plot data for one or more traits assessed in multi-environment field trials (Werner et al. 2024). FieldSimR which is a R package allows the plot error to capture, a spatial trend, random error, as well as extraneous variation e.g., from rows or columns. Phenotypes can be generated by combining the plot errors with genetic values e.g., true, simulated, or predicted. FieldSimR has the capacity to simulate correlated plot errors for multiple traits. The correlation matrix between traits can be set for the spatial, random, and extraneous errors terms separately. FieldSimR provides wrapper functions to simulate correlated genetic values that capture G*E interaction effects with the R package AlphaSimR.

Among the above mentioned simulation tools that are suitable for the evaluation of statistical properties of new statistical methods, QuantiNemo, MaCS, as well as PedigreeSim have been used so far most frequently. The functionality, properties, and assumptions of crop growth models have been recently summarized by Gavasso-Rita et al. (2024) and, thus, will not be included in our review.

## Challenges and limitations

Despite their potential, several challenges and limitations need to be considered when executing and interpreting the results of computer simulations in a plant breeding context. Phenotypes for complex traits are influenced by complex biological processes including complex gene networks with regulation on various levels resulting in epistatic and G*E interactions. Capturing the entire complexity in simulations is in many cases not possible. Therefore, computer simulations often rely on simplifying assumptions to make computations feasible. This can compromise accuracy leading to potentially misleading predictions. This is especially the case, if no or only low quality estimates are available for essential parameters determining the computer simulations. Therefore, experimental data are essential to provide parameters for computer simulations.

As with any scientific result, findings from computer simulations also require validation. Due to the long duration of breeding cycles, a validation of simulation results by experimental results or even real-world breeding outcomes is often not possible. Nevertheless, validation by the use of alternative simulation approaches, sensitivity analyses, or cross-validation is recommended.

Simulating large, realistic breeding populations with detailed genetic and environmental interactions requires substantial computational power and sophisticated algorithms. This complexity can limit the scale and speed of simulations, and thereby restricting their practicality for routine breeding decisions. Researchers often face trade-offs between model complexity and computational effort, sometimes needing to simplify models to run them within reasonable time frames.

## Summary and outlook

Plant breeding programs are complex enterprises that require numerous interdependent decisions. In our view, this complexity is increasing even further with technological innovations becoming available in the various crops. Therefore, experimental evaluation of breeding programs as a whole is nearly impossible. Hence, computer simulations are and will become in the future an even more important tool for the optimum design of breeding programs. As illustrated by this review, many simulation tools with different properties are available so that it is expected that for most of the scenarios to be examined a suitable tool should be available.

**Table 1:** Core facts of computer simulations tools grouped according to their main area of application and sorted in chronological order but clustering related tools

Name of tool	References	Programming language of tool	Operating system	Availability	Restrictions in ploidy	Genetic architecture of trait
*Breeding methodological considerations and Evaluation of statistical properties of new methods*						
QU-GENE Engine	Podlich and Cooper (1998)	Fortran	Windows	https://sites.google.com/view/QU-GENE/Download-page	Diploid	Additive, dominance, and epistasis genetic effects can be simulated Allows the simulation of genotype*environment (G*E) interactions Pleiotropy is considered
QuLine Breeding module	Wang and Dieters (2008)	Fortran	Windows		Diploid	
QuLinePlus Breeding module	Hoyos-Villegas et al. (2019)	Fortran	Linux, macOS, Windows		Diploid	
QuHybrid Breeding module	Wang (2008a)	Fortran	Windows		Diploid	
QuMARS Breeding module	Li (2011)	Fortran	Windows		Diploid	
QMSim	Sargolzaei and Schenkel (2009)	C++	Linux, Windows	https://animalbiosciences.uoguelph.ca/~msargol/qmsim/	Diploid with considering sex chromosomes	Additive QTL effects can be simulated with different distributions, such as gamma, normal or uniform Polygenic effects can be included
BreedingScheme Language (BSL)	Yabe et al. (2017)	R package with C/C++	Linux, macOS, Windows	CRAN, but cannot be installed in R 4.3	Diploid	Additive, dominance, and epistatic effects No G*E interactions can be simulated
ADAM-plant	Liu et al. (2019)	Fortran 95	Linux 32 and 64 bit	The website given in the manuscript cannot be accessed: http://adam.agrsci.dk/	Diploid	Additive effects can be considered G*E interactions are possible to be considered Multiple traits can be simulated that are, if necessary correlated by shared QTL
ADAM-multi	Chu and Jensen (2025)			Contact corresponding author	Diploid and polyploid (allo and auto)	Additive, dominance, and epistatic effects can be considered Multiple traits can be simulated that are, if necessary correlated by shared QTL
MoBPS	Pook and Schlather (2020)	R package	Linux, macOS, Windows	CRAN	Diploid organisms with the possibility of a sex chromosome	Additive, dominance, and epistatic effects Simulation of G*E interactions are possible Multiple traits and correlated traits are possible Other fixed effects can be incorporated
MoBPSweb	Pook et al. (2021)	java-script	Not relevant	Register in the website https://www.mobps.de/, and run in the virtual machine	cf. MoBPS	cf. MoBPS
AlphaSimR	Gaynor and Cgorjanc (2021)	R package with C++	Linux, macOS, Windows	CRAN	Diploid, polyploid (allo and auto), and organisms with sex chromosomes	Additive, dominance, epistatic, and G*E interaction effects possible Note: Dominance effects in autoploid species are modeled by digenic form Epistatic effects are modeled as additive by additive epistatic effects between discrete pairs of loci. G*E interactions are modeled as additive effects whose value is a function of a single environmental covariate. The simulation of multiple traits is possible
XSim version 2 (XSimV2)	Chen et al. (2022)	Julia	Linux, macOS, Windows	https://reworkhow.github.io/XSim.jl/	Diploid and allopolyploid	Additive effects
Blib	Zhang et al. (2022)	Fortran	Windows	Compiled codes for four scenarios available as material of the manuscript	Diploid	cf. QU-GENE Additive, dominance, and epistasis genetic effects Allows the simulation of G*E interactions Pleiotropy is considered
ChromaX	Younis et al. (2023)	Python library	Linux, macOS, Windows	https://chromax.readthedocs.io/en/latest/	Diploid	Additive effects G*E interactions Faux et al. (2016) Dominance and epistasis effects cannot be simulated
SNPcan breeder	Degen and Müller (2023)	Visual Studio 2019 as a .NET application	Windows	https://www.thuenen.de/en/institutes/forest-genetics/software/SNPcan	Diploid sets of chromosomes and co-sexual individuals (monoecious or hermaphroditic)	Genetic architectures: additive effects with considering inbreeding depression during the generations of phenotypes G*E interaction effects are not included The proportions of biallelic, tri-allelic, and tetra-allelic SNP as well as the distribution of frequencies of the common alleles at the SNP can be defined
Python Breeding Optimizer and Simulator (PyBrOpS)	Shrote (2024)	Python	Installation guide available for linux, but should be platform independent	https://rzshrote.github.io/pybrops/	The manual implies that users can decide the ploidy level. But the coding for generation of additive and dominance effects is based on diploid and without considering the process of meiosis in autopolyploid species	Additive and dominance effects Epistatic and non-linear effects are not considered, but they may be included by custom models Multiple traits can be simulated
*Evaluation of statistical properties of new methods*						
QuantiNemo	Neuenschwander et al. (2008)	C++	Linux, macOS, Windows	http://www2.unil.ch/popgen/softwares/quantinemo	Diploid	Additive, dominance, and epistatic effects
QuantiNemo 2	Neuenschwander et al. (2019)	C++	Linux, macOS, Windows	http://www2.unil.ch/popgen/softwares/quantinemo		
Markovian Coalescent Simulator (MaCS)	Chen et al. (2009)	C++	Linux, macOS, Windows	https://github.com/gchen98/macs	Diploid	Not relevant
PedigreeSim	Voorrips and Maliepaard (2012)	Java	Linux, macOS, Windows	https://github.com/PBR/pedigreeSim/	Diploid and polyploid (allo and auto)	Not relevant
PopVar	Mohammadi et al. (2015)	R package	Linux, macOS, Windows	CRAN	Diploid	Additive effects can be considered
XSim	Cheng and Garrick (2015)	C++ & Julia	Linux, macOS, Windows	Julia: https://github.com/reworkhow/XSim.jl C++: https://github.com/reworkhow/XSim.cpp	Diploid	Not relevant
Sequence-Based Virtual Breeding (SBVB)	Pérez-Enciso et al. (2017)	Fortran	Linux, macOS	https://github.com/miguelperezenciso/SBVB	Diploid	Additive and dominance effects Epistatic effects between pairs of loci can be simulated Any number of complex phenotypes that are determined by any number of causal loci
polyploid Sequence Based Virtual Breeding (pSBVB)	Zingaretti and Monfort (2019)	Fortran	Linux, macOS	https://github.com/lauzingaretti/pSBVB	Diploid, polyploid (allo and auto)	Additive and dominance effects are possible. Epistasis is not foreseen Any number of complex phenotypes that are determined by any number of causal loci can be simulated G*E interactions are not considered
GeneEvolve	Tahmasbi and Keller (2017)	C++	Linux, macOS, Windows	https://github.com/rtahmasbi/GeneEvolve	Diploid	Additive and dominance effects
SeqBreed	Pérez-Enciso et al. (2020)	Python 3	Linux, macOS, Windows	https://github.com/miguelperezenciso/SeqBreed	Diploid and polyploid (allo and auto) genomes, as well as sex and mitochondrial chromosomes	Additive and dominance effects Epistatic effects and G*E interactions is not considered Any number of complex phenotypes that are determined by any number of causal loci can be modeled
FieldSimR	Werner et al. (2024)	R package	Linux, mac OS, Windows	CRAN	Not relevant	Not relevant
